# The State of Play on COVID-19 Vaccination in Pregnant and Breastfeeding Women: Recommendations, Legal Protection, Ethical Issues and Controversies in Italy

**DOI:** 10.3390/healthcare11030328

**Published:** 2023-01-22

**Authors:** Roberto Scendoni, Piergiorgio Fedeli, Mariano Cingolani

**Affiliations:** 1 Department of Law, Institute of Legal Medicine, University of Macerata, 62100 Macerata, Italy; 2 School of Law, University of Camerino, 62032 Camerino, Italy

**Keywords:** COVID-19 vaccine hesitancy, pregnant woman, breastfeeding woman, maternity, legal protection, vaccination damage, wrongful birth, indemnity, medical ethics

## Abstract

To date, extensive research has been conducted on vaccination against COVID-19 during pregnancy to verify the safety and efficacy of the vaccines, despite the fact that pregnant women were excluded from the initial clinical trials. The ever-increasing number of scientific publications has confirmed the absence of biological mechanisms associating mRNA vaccines with adverse effects in pregnancy and breastfeeding, although few studies have been carried out on their effect on fertility. While the Italian legal system provides for maternity protection measures and indemnity for vaccination damages pursuant to law no. 210/1992, it is not exempt from controversy. This contribution describes the state of play on COVID-19 vaccination in pregnant and lactating women, including: current recommendations for pregnant and lactating women; ethical issues related to vaccination hesitancy among pregnant women; the legislative paradox whereby sanctions may be imposed on women in certain professional categories who refuse vaccination because they are pregnant; and the possible legal consequences in the event of harm to the unborn child due to vaccination. All of this is considered in accordance with the principles of medical ethics, taking into account the national legislation.

## 1. Introduction

The lack of conclusive data on the safety and efficacy of COVID-19 vaccines for pregnant and breastfeeding women has been a cause of concern among these target populations. Indeed, this has been a topic of much debate in Italy and around the world. Most countries initially recommended that vaccination should be offered to breastfeeding mothers and pregnant women who are at higher risk of exposure to the virus (e.g., healthcare professionals) or at greater risk of developing a severe illness (women who have pre-existing conditions, are obese or come from countries with high immigration rates). Recommendations included individual risk-benefit assessments, which require consultations with healthcare professionals.

In Italy, during the first wave of the pandemic, the Italian Obstetric Surveillance System (ItOSS) of the Higher Institute of Health (ISS) examined interim national and international recommendations, reviewed the relevant scientific evidence and published documents that were endorsed by the main scientific associations in the sector, including the Italian Society of Gynecology and Obstetrics (SIGO), the Italian Society of Neonatology (SIN), the Italian Society of Perinatal Medicine (SIMP) and the Italian Society of Pediatrics (SIP) [1]. 

The first interim document was published on 9 January 2021 and updated on 31 January 2021 [2], after the AstraZeneca vaccine was introduced to the market. A few months later, on 22 September 2021, the ISS revised the recommendations in its original document with a view to help healthcare professionals and pregnant and breastfeeding mothers make informed decisions during the COVID-19 pandemic [3].

Another key date was 13 December 2021, when the ISS further updated the interim indications by recommending that women in the second or third trimester of pregnancy who wished to be vaccinated should be offered a dose of mRNA vaccine, as a booster after a primary vaccination cycle—in compliance with the regulation in force. This recommendation was made on the basis of growing evidence that vaccination in pregnancy was safe, both for the fetus and for the mother. It also took into consideration evidence relating to: (i) the increased risk of morbidity from infection with the Delta variant; (ii) the increasing transmission rates at that time; and (iii) the significant decrease in the median age of infection in Italy.

On 7 October 2022, the ItOSS once again updated its guidelines on vaccination against COVID-19 during pregnancy and breastfeeding, in light of the new vaccines available and the introduction of the second booster shot (fourth dose) for this group [4].

## 2. Data on Vaccination Efficacy and Safety in Pregnant Women

SARS-CoV-2 infection during pregnancy represents an autonomous risk factor, with an increase in ICU admissions and maternal mortality rates; equally higher is the rate of preterm births, stillbirths, caesarean sections and other diseases related to pregnancy [5,6,7]. 

Taking for granted the specific risk to the health of the pregnant woman who contracts a SARS-CoV-2 infection, based on the current availability of numerically consistent data, international public health agencies argue that primary vaccination plus third and fourth booster doses offer the safest and most effective way to protect pregnant women and their unborn babies from COVID-19, thanks to the transplacental transfer of maternal antibodies to the fetus, which mainly occurs in the last weeks of pregnancy [8]. In September 2022, the Italian Ministry of Health recommended bivalent formulations of the mRNA vaccines Comirnaty Original/Omicron and Spikevax Original/Omicron as a booster dose (fourth dose) for pregnant or breastfeeding women, along with fragile populations, after completing a primary course of COVID-19 vaccination [9].

Experience with other vaccinations suggests that COVID-19 vaccines may be equally effective in pregnant and non-pregnant women. Indeed, there is growing (if not conclusive) evidence on the immunogenicity, safety and efficacy of vaccines against COVID-19 in pregnancy (this type of data was previously unavailable, since the first clinical trials did not involve pregnant women, as mentioned earlier). For instance, a retrospective cohort study in Israel including pregnant women who received mRNA vaccines in 2021 reported a significantly lower risk of contracting a SARS-CoV-2 infection, compared to unvaccinated women [10].

Other studies have also described a reduction in the incidence of SARS-CoV-2 infections in pregnant vaccinated women [11]; however, these were observational studies, often involving a small sample of women, therefore the findings cannot be considered conclusive.

The largest case series on the safety profile of mRNA vaccines, published by Shimabukuro et al. in the *New England Journal of Medicine* (2021) [12], surveyed more than 35,000 women. However, only 827 participants had a completed pregnancy and findings did not show clear safety signals among pregnant persons who received mRNA COVID-19 vaccines. Other observational studies, with smaller groups of subjects, have found no differences in the post-vaccination symptoms reported by pregnant and non-pregnant women [13]. 

As regards gestational age, there is no conclusive evidence about the optimal window for vaccination, because few women to date have received the vaccine in the first trimester of pregnancy. 

In a cohort of 1328 women who gave birth in the UK between March 2020 and July 2021, only 140 were found to have received at least one dose of the vaccine (mostly during the third trimester of pregnancy). Unvaccinated women are more frequently socially disadvantaged and foreign. Researchers in the US have compared the rate of adverse events (stillbirth, fetal abnormalities, cesarean section, small for gestational age newborns, admission to maternal intensive care unit or neonatal intensive care unit and prematurity) in 131 vaccinated and 393 unvaccinated pregnant women—on the basis of a propensity score that included an index of multiple deprivation (IMD) score, maternal age and drug use: no differences between the two groups were found in any of the outcomes considered [14]. In the UK, following more than 200,000 vaccinations during pregnancy, no adverse effects greater than those in the non-pregnant population were reported [15].

A recent study has demonstrated that a third dose of the BNT162b2 mRNA COVID-19 vaccine during pregnancy, given at least five months after the second vaccine dose, enhances protection against adverse COVID-19-related outcomes [16]. Furthermore, there is no evidence of reduced fertility in women after vaccination [17,18].

A recent systematic review and meta-analysis on the COVID-19 vaccine and pregnancy outcomes [19] concludes: “The probability of small for gestational age is similar between vaccinated and unvaccinated pregnant women, and the former also had a slightly reduced rate of premature delivery”.

Some researchers have demonstrated the presence of antibodies against the SARS-CoV-2 in cord blood and breast milk in response to infection during pregnancy, suggesting a possible passive immunity in the newborn [20]. The transfer of antibodies through the umbilical cord has also been reported following administration of mRNA vaccines [21], as mentioned at the beginning of this section. Although breastfeeding women have not been included in studies evaluating vaccines against COVID-19, the effectiveness of vaccination is believed to be similar to that found among non-pregnant women [22]. Thanks to the demonstration of the presence of anti SARS-CoV-2 antibodies in the milk of vaccinated women, it is conceivable that the newborn acquires additional protection against a SARS-CoV-2 infection, even if the degree of this protection is not yet known [23].

Another systematic review demonstrates that vaccination against COVID-19 in pregnant and lactating women is immunogenic and does not cause relevant vaccine-related adverse events [24].

## 3. Current Recommendations for Pregnant and Breastfeeding Women in Italy

There are multiple recommendations for the use of mRNA vaccines in pregnant women; fewer are the restrictions on the use of vaccines with viral vectors for breastfeeding. Many European countries, along with the United States and the United Kingdom, have remained in the same decision-making position for pregnancy (Table 1) and breastfeeding (Table 2) [25].

In Italy, mRNA vaccines have been particularly effective in preventing severe COVID-19 in pregnant women. The actual policy position for vaccination in Italy, both in pregnant and lactating women is shown in Table 3 [20]. 

ItOSS surveillance data demonstrated a significant reduction in the risk of severe COVID-19 defined as interstitial pneumonia associated with the need for ventilatory therapy and/or ICU admission—among vaccinated versus unvaccinated cohorts of women with a SARS-CoV-2 infection hospitalized between 1 January and 31 May 2022 [26]. The data confirm an increased risk of developing serious disease for unvaccinated women, which underlines the importance of public health recommendations to promote vaccination [27].

In accordance with and in support of the provisions of the circular of the Ministry of Health of 7 September 2022 [9], the ItOSS-ISS released new recommendations for pregnant and breastfeeding women, including the following points:

- Primary vaccination against COVID-19 and booster doses (third and fourth doses) with mRNA vaccines are recommended for all pregnant women at any stage of pregnancy, especially if there is an increased risk of developing severe COVID-19 disease, as well as for all breastfeeding women, without any need to stop breastfeeding;
- There must be an interval of at least 120 days between administration of the booster dose and the last previous dose of an anti-COVID-19 vaccine or previous SARS-CoV-2 infection;
- The primary vaccination course and booster doses (third and fourth doses) can be administered at the same time as the recommended vaccinations against influenza and pertussis during pregnancy;
- Primary vaccination and booster doses (third and fourth doses) with mRNA vaccines do not expose infants to risks, but rather allow them to acquire antibodies against SARS-CoV-2 through the mother’s milk;
- The vaccination schedule for a newborn baby breastfed by a vaccinated mother does not require any modification.

## 4. Controversies Regarding Vaccination in Pregnant Women

### 4.1. COVID-19 Vaccine Damage: Legal Protection Provided in Italy

Vaccination is a health treatment which, according to the Constitutional Court, has a dual purpose: on an individual level, it aims to protect the person who receives the vaccination, while on a collective level it aims to protect others by helping to stop the infectious disease from spreading. In Italy, COVID-19 vaccinations are compulsory for the entire population of over-50s and for some professional categories.

In the event of vaccine complications, Italian legislation provides for the protection of the injured party in two possible ways: indemnity (“indennizzo”) and compensation for damages (“risarcimento”).

In the Italian legal system, indemnity is regulated by law no. 210 of 1992 [28], that it does not require the existence of profiles of guilt. In other words, in no-fault compensation, the indemnifiable damage is not attributable to culpable conduct assumed by any subject involved in the vaccination procedure but arises from the mere occurrence of the irreversible impairment directly caused by the vaccination. Pursuant to the provisions of paragraph 1 bis of art. 1 of law no. 210/1992, introduced with Legislative Decree no. 4 of 27 January 2022 [29], “indemnity is also due to those who have suffered injury or disability resulting in a permanent impairment of psycho-physical integrity, due to the anti SARS-CoV-2 vaccination recommended by the Italian health authority”. Indemnity is also provided for non-compulsory categories possibly affected.

On the one hand, the right to indemnity disregards the fault and does not require proof of an offense, arising solely from ascertainment that the irreversible impairment is a direct consequence of the vaccination; on the other hand, compensation for damage, pursuant to the provisions of art. no. 2043 of the Italian civil code [30], assumes that the damage is attributable to culpable or willful conduct by the Pharmaceutical Company, the attending physician, the vaccinator, the Ministry of Health or other subjects involved in the procedure of administration or production of the vaccine.

### 4.2. Medical Ethical Issues Concerning the Vaccination of Pregnant Women

Healthcare professionals should give their patients evidence-based advice about vaccination, to help people make an informed decision about receiving the COVID-19 vaccination.

Informed consent is a fundamental component of the ethical-clinical management of a pregnancy [31]. As with all forms of medical therapy, informed consent should precede the administration of a COVID-19 vaccine during pregnancy. In the discussion of informed consent, healthcare professionals (gynecologists, general practitioners, etc.) have a responsibility to provide key information for decision-making, including indications, vaccine benefits and risks, and available alternatives. Healthcare professionals should also advise patients about the potential repercussions of not vaccinating, highlighting the risks for themselves, close contacts and the general population. This means respecting the patient’s autonomy while at the same time promoting the patient’s well-being.

In this context, it is necessary to highlight the issue of non-inclusion of pregnant women in clinical studies or inclusion requirements for women to use contraception before and during treatment for COVID-19 [32].

From the substantial absence of the experimentation phase on these subjects derives a decidedly significant lack of information on the potential risks/benefits for pregnant women and on the product of conception; information gap all the more relevant in the early stages of the vaccination campaign, when prospective studies on vaccination in pregnancy were absent.

From the substantial absence of the experimentation phase on these subjects derives a decidedly significant lack of information on the potential risks/benefits for pregnant women and on the product of conception; information gap is all the more relevant in the early stages of the vaccination campaign, when prospective studies on vaccination in pregnancy were absent.

This exclusion from clinical trials, linked to the frail condition of pregnant and breastfeeding women and to the potential risks for the fetus, could be motivated by the risks that pharmaceutical companies could face, in the event of adverse events in the course of experimentation; in any case, there has been an inequality in health care for this population. In the absence of evidence-based experimental studies, pregnant women have been forced to make decisions about vaccination without having reliable data on the risks to themselves and to the fetus. In other words, pregnant and breastfeeding people have not had the same guarantees from clinical research as the rest of the population [33]. 

This is an issue that also has important repercussions on the behavior of health professionals who have to give advice to pregnant women on the choice of getting vaccinated.

It is well known that health professionals strongly influence patients’ decisions to accept vaccination [34]. If a pregnant patient remains unsure about vaccination, obstetrician-gynecologists or clinicians should inquire about the reasons for this hesitation to help address the individual’s specific questions and concerns [35]. During follow-up visits, obstetrician-gynecologists should deal with ongoing questions and concerns and offer the vaccination again if the woman appears to be willing. In these scenarios, healthcare professionals have the opportunity to implement alternative strategies to protect patient and community health at large, provide patients with instruction on monitoring and managing symptoms at home, and recommend behavioral approaches to reduce associated the risks of SARS-CoV-2 infections and transmission.

The reasons why some pregnant women lack confidence may be related to exposure to misinformation about vaccination efficacy and safety on social media, which has allowed for the widespread dissemination of myths and inaccurate information that further fuels the anti-vaccination movement [36]. 

Healthcare professionals have an ethical obligation to their individual patients and to society to follow evidence-based guidelines by encouraging patients to get vaccinated and to get vaccinated themselves. However, if the pregnant woman refuses the recommended vaccination, her choice must be respected; this was all the more true in the early stages of the SARS-CoV-2 pandemic when, given the exclusion from vaccine trials and the absence of prospective data, this information could not be provided to pregnant women in highly reliable terms; a problem that must be a stimulus for states and scientific societies to set up operational paths.

To the state, the recommendations developed in Italy on anti-COVID vaccination in pregnant women are ethically consistent with the indications provided in the ACOG Committee Opinion, Ethical Issues with vaccination in Obstetrics and Gynecology. As healthcare professionals, gynecologists have an ethical and deontological duty to promote protection from infectious diseases, in accordance with the updated clinical guidelines relating to vaccines. They should therefore counsel their patients about vaccination in an evidence-based way that allows patients to make an informed decision. Moreover, if a pregnant or breastfeeding woman shows hesitancy or reluctance to be vaccinated, specialists should address questions and concerns and re-proposing vaccination at a later stage if recommended.

Gynecologists should explain to their pregnant and breastfeeding patients the safety and efficacy of the COVID-19 vaccination with a particular communicative approach, entering into empathy and understanding of the woman’s emotional state, not exempting themselves from communicating the possible generic complications related to vaccine administration [37]. An adequate assessment of the person’s condition should always be carried out; In fact, many pregnant or breastfeeding women are often psychologically vulnerable and require particular attention in being guided on important and conscious choices, such as vaccination [38].

### 4.3. The Legislative Paradox

Art. no. 4, paragraph 1, of legislative decree no. 44 of 1 April 2021 [39], “in order to protect public health and maintain adequate safety conditions in the provision of treatment and assistance services”, established that free vaccination for the prevention of SARS-CoV-2 infections constitutes an essential requirement for the lawful exercise of certain professions, obliging the vaccination, under penalty of suspension: pharmacists, doctors, dentists, veterinarians, biologists, physicists, chemists, psychologists, nurses, midwives, health, rehabilitation and prevention technicians. The obligation was subsequently extended to teachers and the police.

Vaccines have a high social value, because in addition to protecting the vaccinated person, they reduce the risk of contagion to the rest of the population, as stated earlier. Vaccination is therefore a continuous balance between the individual and collective dimensions of health in the spirit of mutual solidarity between the individual and the community [40]. 

The only exemption to the vaccination requirement is provided for by paragraph 2 of art. no. 4, only for hypotheses of ascertained danger to health, in relation to specific documented clinical conditions, certified by the general practitioner. Of course, it was problematic for pregnant women belonging to one of the aforementioned professional categories who refused the COVID-19 vaccination, many of whom were suspended from employment as they were unable to exploit their condition as a means of obtaining exemption. This also resulted in the loss of maternity benefits, according to the national legislation in force [41]. 

This legislation would not protect the rights of pregnant or breastfeeding women. On the one hand, they are considered fragile subjects, and as such should not be included in experimental studies for vaccines and treatments for COVID-19; on the other hand, they are treated as equivalent to the rest of the population and, as such, subject to the vaccination obligation with the application of sanctions in case of refusal.

It is believed that the provision of sanctions for unvaccinated pregnant women can only be accepted when, following prospective studies, the non-dangerousness and usefulness of vaccination during pregnancy have been ascertained. Moreover, in the initial stages of the vaccination campaign, in the absence of data relating to trials on pregnant women, we believe it was incorrect to provide for compulsory vaccination also for pregnant women. This theme will have to be the subject of discussion and prediction of operating models, to adopt the right behavior in the ethics of a job well done perspective [42].

Ideally, policymakers should use less intrusive means or methods to encourage voluntary vaccination against COVID-19 before contemplating mandatory vaccination. Efforts should be made to demonstrate the health risks of not vaccinating and the benefits and safety of vaccines for the greatest possible uptake of vaccination, especially in pregnant or lactating women. As with other public health policies, decisions about mandatory vaccination should be supported by the best available evidence and made by legitimate decision makers in a transparent, fair, equitable and non-discriminatory manner [43]. In particular, pregnant women or women who have recently given birth often have emotional lability. Therefore, particular attention should be paid not to undermine the mental health of the person who even temporarily refuses a vaccination that is considered to be a mandatory condition for continuing to work in the same category of employment.

In the national regulatory system [41], the territorial labor inspectorate and local health authority provides for early abstention from work for pregnant workers up to the period of compulsory abstention for the following reasons:

(a) In the event of serious pregnancy complications or persistent morbid conditions which are presumed to be aggravated by the state of pregnancy;
(b) When the working or environmental conditions are deemed prejudicial to the health of women and children;
(c) When the worker cannot be transferred to other tasks.

In this context, a possible solution could be to include cases of pregnancy of personnel obliged to be vaccinated in the field of early abstention from work, due to exposure to risk from prejudicial working conditions, as explained in point (b).

### 4.4. What Are the Potential Legal Consequences in the Event of Harm to the Unborn Child due to Vaccination? 

Concerns about the effect of vaccination on the fetus should be discussed in light of relevant medical evidence and understood in the context of each patient’s broad social network, cultural beliefs and values. The effort to emphasize the fetal and neonatal risks of non-vaccination has been reported to be an effective strategy for overcoming vaccine hesitancy among pregnant and lactating patients.

Based on current reports, it is reasonable to propose vaccination to all pregnant women; however, given that relevant studies are still ongoing or nearing completion, it is equally reasonable to consider vaccination cautiously during the first trimester of pregnancy, when hyperpyrexia can be associated with a significant, albeit limited, increased risk of congenital malformations, such as neural tube defects [17,44].

In the event of ascertained damage to the fetus correlated with vaccination of the pregnant woman, Italian legislation recognizes damage from “wrongful birth”.

In common law, as in many civil law countries, the term wrongful birth refers to an unintentional birth that occurs as a result of medical malpractice or a failure in some form of birth control procedure [45]. Claims of illicit birth are often based on failure to warn that a child will be born with serious health problems. The doctor negligently failed to disclose to the prospective parents the risk of having a child with a congenital disease; so, the parents were deprived of choosing to terminate the pregnancy in case of awareness of the possible damage to the child. 

In such cases, the parents are entitled to request compensation [46]. There is absolute agreement on this point. The damage must obviously be demonstrated according to the general rules and can fall into one of two categories:

- Non-pecuniary damage: this is the so-called psychic damage suffered by parents having to face a new, unforeseen situation of malformation or disease in their child. Obviously in this case, the existence of psychic damage must be demonstrated;
- Pecuniary damage: consisting of additional expenses incurred in order to properly care for and meet the needs of the malformed child.

The reasoning is based on the assumption that the mother has the legal freedom to interrupt the pregnancy whenever she wishes, on the basis of a prenatal diagnosis, when there is reason to believe that serious damage to her physical or psychological health may result from the birth.

The question of the malformed child’s right to compensation is much debated. The prevalent view is that the child born malformed has no right to claim compensation for wrongful birth damage [47]. This thesis, however, is not unanimously agreed upon.

Recently, an alternative line of thought has emerged according to which the unborn child would have not only the right to be born healthy but also the right not to be born unhealthy (malformed due to an incorrect prenatal diagnosis which therefore prevented the mother from exercising her right to terminate the pregnancy). Such rights are obviously decided by the mother if she is correctly informed about the conditions of the fetus.

According to this approach, it follows that the subject born with complications or malformations would also have the right to request and obtain compensation for damages.

## 5. Conclusions

COVID-19 vaccination coverage remains lower among pregnant individuals than among non-pregnant women of reproductive age [48]. Given the risks of severe disease and adverse pregnancy outcomes, it is imperative to continue to collect and disseminate data on the safety and efficacy of COVID-19 vaccination in pregnancy and to encourage healthcare professionals to provide the necessary information and to promote vaccination where there are no clinical shortcomings or impediments. Whether pregnant or breastfeeding, the woman is experiencing a particular moment in her life, therefore it is always necessary to safeguard, not only the physical, but also the mental health of the person concerned, considering all of the eventualities and exceptions of the single case in terms of COVID-19 vaccination. Aspects of medical ethics cannot be overruled by legislative obligations. It is hoped that this overview of the state of play in Italy on COVID-19 vaccination in pregnant and lactating women may help systematize processes that could be implemented to protect the health of this population; it could be useful in other endemic or pandemic situations that might occur in the future. Indeed, the experience of the COVID-19 pandemic signals the need for any recommendations drawn up in the event of future pandemics to be based on validated scientific knowledge concerning vaccines and on a correct assessment of the risk–benefit ratio in the particular case of their administration in pregnancy. A limitation of this report is that the decision-making position is constantly evolving, taking into account the trend of the pandemic; the above illustrates the situation updated in January 2023. Furthermore, the legal consequences are also not well defined; many verdicts on the impairments of COVID-19 vaccination will only be issued in a few months or years.

## Figures and Tables

**Table 1:** Actual policy position for vaccination during pregnancy, by country (Europe, United Kingdom, United States), for each vaccine.

Country	Pfizer/BioNTech Comirnaty	Moderna COVID-19 Vaccine	Oxford-AstraZeneca Vaxzevria, Covishield	Johnson & Johnson Janssen COVID-19 Vaccine	Sinopharm BIBP-CorV	Gamaleya Sputnik V	Novavax Covovax, TAK-019
Austria							
Belgium							
Bulgaria							
Croatia							
Cyprus							
The Czech Republic							
Denmark							
Estonia							
Finland							
France							
Germany							
Greece							
Hungary							
Ireland							
Latvia							
Lithuania							
Luxembourg							
The Netherlands							
Poland *							
Portugal							
Romania							
The Slovak Republic							
Slovenia							
Spain							
Sweden							
Switzerland							
The United Kingdom							
The United States							

**Table 2:** Actual policy position for vaccination during lactation, by country (Europe, United Kingdom, United States), for each vaccine.

Country	Pfizer/BioNTech Comirnaty	Moderna COVID-19 Vaccine	Oxford-AstraZeneca Vaxzevria, Covishield	Johnson & Johnson Janssen COVID-19 Vaccine	Sinopharm BIBP-CorV	Gamaleya Sputnik V	Novavax Covovax, TAK-019
Austria							
Belgium							
Bulgaria							
Croatia							
Cyprus							
The Czech Republic							
Denmark							
Estonia ^+^							
Finland							
France							
Germany							
Greece							
Hungary							
Ireland							
Latvia							
Lithuania							
Luxembourg							
The Netherlands							
Poland *							
Portugal							
Romania							
The Slovak Republic							
Slovenia							
Spain							
Sweden							
Switzerland							
The United Kingdom							
The United States							

Legends: 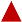 Not recommended; 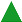 recommended; 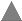 no position found; 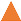 not recommended but with exceptions; 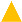 permitted with qualifications; 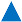 permitted; * vaccination permitted, but the type of vaccine is not specified; + vaccination recommended, but the type of vaccine is not specified; 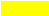 countries that have different recommendations for vaccination during lactation versus vaccination during pregnancy.

**Table 3:** Actual policy position of the Public Health Authorities for vaccination during pregnancy and lactation in Italy.

Public Health Autority	Pfizer/BioNTech Comirnaty (0.3 mL)	Moderna COVID-19 Vaccine (0.5 mL)	Oxford-AstraZeneca Vaxzevria, Covishield (0.5 mL)
	Pregnancy		
Superior Institute of Health (ISS)			
Ministry of Health			
Italian Society of Gynecology and Obstetrics			
	**Lactation**		
Superior Institute of Health (ISS)			
Ministry of Health			
Italian Society of Gynecology and Obstetrics			

## Data Availability

The study did not report any data of patients.
